# Electronic Structure and Chemical Bonding in Methylammonium Lead Triiodide and Its Precursor Methylammonium Iodide

**DOI:** 10.1021/acs.jpcc.2c06782

**Published:** 2022-11-17

**Authors:** Cody M. Sterling, Chinnathambi Kamal, Alberto García-Fernández, Gabriel J. Man, Sebastian Svanström, Pabitra K. Nayak, Sergei M. Butorin, Håkan Rensmo, Ute B. Cappel, Michael Odelius

**Affiliations:** † Department of Physics, Stockholm University, AlbaNova University Center, SE-106 91Stockholm, Sweden; ‡ Theory and Simulations Laboratory, Theoretical and Computational Physics Section, Raja Ramanna Centre for Advanced Technology, Indore452013, India; § Homi Bhabha National Institute, Training School Complex, Anushakti Nagar, Mumbai400094, India; ∥ Division of Applied Physical Chemistry, Department of Chemistry, KTH - Royal Institute of Technology, SE-100 44Stockholm, Sweden; ⊥ Condensed Matter Physics of Energy Materials, Division of X-ray Photon Science, Department of Physics and Astronomy, Uppsala University, Box 516, SE-75121Uppsala, Sweden; # GJM Scientific Consulting, Fort Lee, New Jersey07024, United States; ∇ Tata Institute of Fundamental Research, 36/P, Gopanpally Village, Serilingampally Mandal, Ranga Reddy District, Hyderabad500046, India

## Abstract

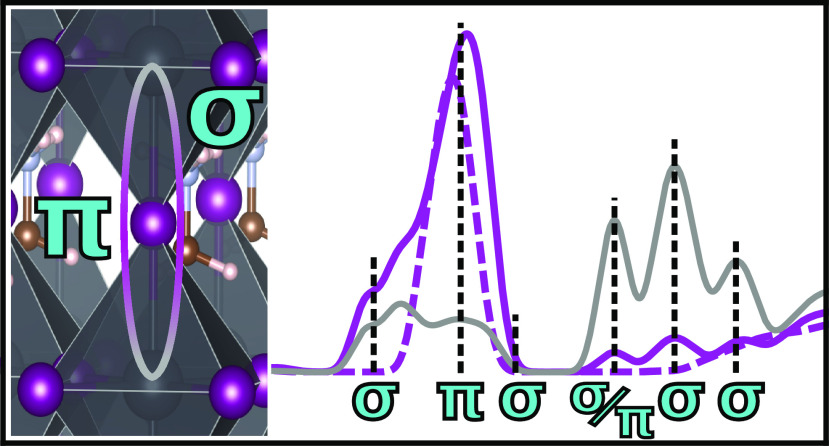

A detailed examination of the electronic structures of methylammonium lead triiodide (MAPI) and methylammonium iodide (MAI) is performed with *ab initio* molecular dynamics (AIMD) simulations based on density functional theory, and the theoretical results are compared to experimental probes. The occupied valence bands of a MAPI single crystal and MAI powder are probed with X-ray photoelectron spectroscopy, and the conduction bands are probed from the perspective of nitrogen K-edge X-ray absorption spectroscopy. Combined, the theoretical simulations and the two experimental techniques allow for a dissection of the electronic structure unveiling the nature of chemical bonding in MAPI and MAI. Here, we show that the difference in band gap between MAPI and MAI is caused chiefly by interactions between iodine and lead but also weaker interactions with the MA^+^ counterions. Spatial decomposition of the iodine p levels allows for analysis of Pb–I σ bonds and π interactions, which contribute to this effect with the involvement of the Pb 6p levels. Differences in hydrogen bonding between the two materials, seen in the AIMD simulations, are reflected in nitrogen valence orbital composition and in nitrogen K-edge X-ray absorption spectra.

## Introduction

In light of growing concerns about climate change due to greenhouse gas emissions from fossil fuel use, research into renewable energy sources is essential. One of the most promising avenues for this is solar power, and in this area, perovskite materials, specifically hybrid organic–inorganic perovskites (HOIPs), have recently received particular attention.^1−3^ Since their debut by Kojima et al.^4^ in 2009 with a power conversion efficiency (PCE) of around 3%, HOIP solar cell PCEs have rapidly increased to over 25% by Jeong et al.,^5^ on par with traditional silicon solar cells.^6^

Despite extensive research into HOIPs, however, key issues still remain. One of these is stability, where PCE can decrease dramatically in relatively short time spans due to, e.g., heat and humidity.^5^ Understanding their (lack of) stability requires insight into the chemical bonding of the HOIPs and associated precursor materials. The exact origin of their optoelectronic properties is also still up for debate.^7^ Naturally, since it is related to the optical absorption efficiency, there is interest in the band gap of this family of materials^8−10^ and how it changes by altering, e.g., the halide used.^11^ Gaining a better understanding of the factors that contribute to the character of the valence band maximum and conduction band minimum is therefore very important in understanding how to tune the band gap to improve solar cell function. In one of the prototypical HOIPs, methylammonium lead triiodide (CH_3_NH_3_PbI_3_, or MAPI), the valence and conduction bands are determined predominantly by the PbI_3_ lattice atoms.^12,13^ The effect of spin–orbit coupling (SOC) has been investigated previously in MAPI and other HOIPs,^14^ showing good performance for electronic structure calculations, based on density functional theory (DFT), using hybrid functionals with the inclusion of SOC (e.g., HSE06/SOC).^15^ It has also been shown that, due to favorable error cancellation, DFT calculations, within the standard generalized gradient approximation (GGA) and neglecting SOC, can yield an appropriate band gap and small differences in valence/conduction band structure compared to SOC calculations.^15,16^ The related Rashba effect was investigated by Etienne et al.^17^ where they found that, though notable in small MAPI cells, this effect is likely negligible in larger MAPI cells due to disordered orientations of the MA^+^ cation at room temperature.

Angular-resolved photoemission spectroscopy (ARPES) is a useful experimental technique to measure electronic energy-momentum dispersion in materials by measuring the energy and momentum information of ejected excited electrons. By changing the angle of incident photons, different bands from chemical bonding in different directions can be probed to give more complete information about the electronic band structure of a material. This has been used in perovskites, for example, to examine the elemental interactions making up the valence and conduction bands. The results of Wang et al. indicate that the valence band of halide HOIPs is formed by the halide p and lead s orbitals.^18^ Another study by Lee et al. in 2017 used ARPES to show that the valence band is made up of antibonding σ interactions between I 5p and Pb 6s orbitals.^19^

In HOIP materials, it is largely unknown to what extent the organic cation affects the optoelectronic properties, particularly in relation to the inorganic lattice.^7^ Our previous computational work on MAPI investigated the effect of different geometric parameters related to MA^+^ on its nitrogen K-edge (N 1s) X-ray absorption (XA) spectra, finding that the ways the organic cation can interact with the inorganic Pb–I lattice affects its XA spectrum and therefore its electronic structure.^20^ Investigations of the subsequent X-ray fluorescence decay and Auger decay channels^21,22^ suggest the appearance of hybridized MA^+^ levels in the (lead-and-iodide dominated) valence band of MAPI. A recent investigation by Ong et al. shows that the band gap in MAPI can change due to the rotation of the MA^+^ cation, mostly by affecting the conduction band.^23^

In this work, we investigate the difference between the electronic structure of MAPI and its precursor methylammonium iodide (CH_3_NH_3_I, or MAI) in more detail, with specific regard to the organic MA^+^ ion and, in MAPI, the inorganic PbI_3_^–^ framework. Previous research on MAI has revealed insight into its crystal structure and orientation of the MA^+^ cation,^24^ and detailed vibrational spectroscopy measurements have been performed over a range of temperatures and orientations.^25^ However, there has been limited detailed research into its electronic structure on an element-specific basis, which can be achieved with a combination of X-ray spectroscopy and quantum chemical calculations.^26^ In both MAPI and MAI materials, the MA^+^ ions can interact with the iodide ions, but MAPI additionally has an inorganic PbI_3_^–^ framework, which is sensitive to the organic cations.^8^ In a recent study, we have shown that the valence and conduction bands of lead bromide perovskites (APbBr_3_) are strongly influenced by interactions with the positive cations at the A-site.^27^

Through a detailed side-by-side comparison of the electronic structure of the MAPI and MAI materials, we seek to isolate and clarify the effects that the Pb–I inorganic lattice and its interactions with the organic MA^+^ ions have on the valence and conduction bands of MAPI. *Ab initio* molecular dynamics (AIMD) simulations of both materials provide representative sampling of MA^+^ orientations in each material, and comparing structural differences in radial distribution functions of sampled geometries allows us to establish distinct differences in the MA^+^ and I^–^ interactions in MAPI and MAI. These structural effects are rationalized on the basis of the local electronic density of states, which reveals related influences in chemical bonding. We investigate the interactions by sampling geometries of AIMD trajectories of the two materials. To investigate and validate the models, the occupied projected density of states of the Kohn–Sham orbitals was compared with experimental X-ray photoelectron spectroscopy (XPS) measurements, and the unoccupied states were explored in calculated and experimental N 1s XA spectra. Following this, we perform an extensive breakdown of the electronic density of states separated into elemental and orbital contributions for MAPI and MAI. This allows us to obtain detailed insight into the effect that the lead-iodine lattice has on the electronic structure of MAPI, both with regard to the iodine levels hybridizing with lead and how iodide levels are influenced by the MA^+^ ions in the conduction and valence bands in MAPI. Our element-specific analysis builds on previous research on the main orbital contributions to the valence band structure of MAPI^13,18,19^ to separate these interactions between σ and π bonds between the iodine and lead states, as well as isolating the effect of the lead on the MA^+^ valence states.

## Methods

### Computational Details

The MAPI and MAI materials were modeled using periodic DFT in the CP2K program suite,^28^ using AIMD simulations on large supercells to derive information about dynamics in the crystal structure and electronic structure—in particular hydrogen bonding, electronic projected density of states (PDOS), and simulated N 1s XA spectra. The computational protocol in this work is largely the same as we have used in previous work on methylammonium lead-halide perovskites.^20,22^

The starting crystal geometries of tetragonal MAPI and MAI were generated according to experimental parameters.^24,29^ These cells were repeated to create supercells of 2 × 2 × 2 and 3 × 3 × 2 crystal unit cells for MAPI and MAI with a total of 32 and 36 MA^+^ molecules, respectively. This results in tetragonal supercells with cell parameters of (*a* = *b* = 17.710 Å, *c* = 25.318 Å) for MAPI and (*a* = *b* = 15.383 Å, *c* = 18.036 Å) for MAI. The initial orientations for all MA^+^ ions were along the *z* axis to provide an ideal crystal starting structure for both systems, and this orientation is also suggested for MAI by Yamamuro et al.^24^ All DFT-based calculations were performed using the Perdew–Burke–Ernzerhof (PBE) exchange–correlation functional^30^ with the Grimme’s D3 van der Waals correction,^31,32^ unless specified otherwise.

The AIMD simulations were performed using the Gaussian and plane wave (GPW) formalism^33^ with a plane wave cutoff of 600 Rydberg in the representation of the electron density. Goedecker–Teter–Hutter (GTH) pseudopotentials^34,35^ were used and basis sets of TZVP-MOLOPT-GTH type for C, N, and H^36^ and of DZVP-MOLOPT-SR-GTH type for I and Pb.^34^ We employed a time-step of 0.5 fs using the NPT ensemble at ambient conditions (300 K and 0 atm) for 50 ps, the first 10 ps of which serve as equilibration. We used a four-chain Nosé–Hoover thermostat^37^ with a coupling time constant of 20 fs, and the barostat had the same coupling with a temperature tolerance of 100 K and employed an isotropic NPT algorithm which preserves the angles and the ratios of cell parameters *a*/*c* and *b*/*c*. We note that the fluctuations in cell parameters and temperature equilibrated after around the first 3 ps of simulation for average MAPI dimensions with a 95% confidence interval of *a* = *b* = 17.9 Å, *c* = 25.6 Å with fluctuations around 0.14–0.2 Å, and for MAI of *a* = 15.4 Å, *b* = 15.6 Å, *c* = 18.1 Å with fluctuations around 0.36–0.42 Å, where the error in the averages are below 0.05 Å.

Snapshots of the cell geometries were sampled for five configurations at 10 ps intervals to ensure uncorrelated samples, which gives sufficient statistics to analyze the electronic structure in terms of sampling of XA spectra and PDOS over the multiple (32 or 36) MA^+^ ions in the supercells. To understand dynamical effects on structural properties, radial distribution functions, *g*(*r*), for C···I, N···I, and H···I distances were also studied. The latter were separated into categories of carbon- and nitrogen-attached hydrogen atoms, denoted (C)H and (N)H, respectively. To evaluate the representative samples, we also compared *g*(*r*) from the sampled configurations and the full trajectories of the two materials. The coordination was analyzed based on cumulative integrals of the *g*(*r*), given as *n*_B_(*R*_AB_) = ∫_0_^*R*_AB_^ ρ_B_*g*(*r*_AB_)4π*r*_AB_^2^d*r*_AB_, where ρ_B_ is the density of atom type B.

The N 1s XA spectrum for each nitrogen in the supercells was calculated using the half core–hole transition potential (TP_HH) approximation in the Gaussian augmented plane wave (GAPW) method.^38^ The XA spectrum simulations were performed using the 6-31++G(2d,2p) basis sets on the MA^+^ atoms,^39,40^ while the description of the inorganic ions and the choice of functional approximation were identical to the AIMD simulations. The spectra were calculated using 1000 added unoccupied orbitals. For evaluation of the electronic structure and chemical bonding, PDOS calculations were performed using CP2K on each of the five sampled configurations still under the GAPW formalism using the 6-31++G(2d,2p) basis set on the MA^+^ atoms, and curves were combined to create averaged curves for MAPI and MAI. The PDOS calculations were performed using the ground-state orbitals, which excludes final-state orbital relaxation effects present in the experimental XPS process.

For comparison of the PDOS and XA calculations to experimental XPS and XA data, the discrete theoretical intensities were convoluted using a normalized Gaussian curve with σ = 0.2 eV (corresponding to a full width at half-maximum of ). In relation to XPS, we note that the energy derivative discontinuity is not well described in DFT with exchange–correlation functionals using the local density approximation and GGA,^41^ and hence ionization energies are not quantitatively reproduced. Thus, an *ad hoc* constant energy shift of −2.68 eV was applied to the Kohn–Sham eigenvalues for comparison to the experimental XPS data of MAPI and −2.49 eV for MAI. An unrelated *ad hoc* constant energy shift of −2.74 eV was applied to simulated XA spectra for both MAPI and MAI, aligned to the main peak of the measured XA spectrum of MAPI. The shift required for the PDOS is mainly due to limitations in basis sets and the functional approximation in DFT, whereas the shift associated with the mismatch in the simulated XA spectra has additional limitations from the transition potential approximation and neglect of relativistic effects. The *ad hoc* energy shifts do not affect the analysis of relative energies and character of orbitals involved in the electronic transitions leading to the peaks in the experimental XPS and XA spectra.

Additionally, to investigate the effect of spin–orbit coupling (SOC), calculations using the Quantum ESPRESSO code^42,43^ with and without SOC were performed on all sampled geometries for MAPI and MAI. As with the CP2K PDOS calculations, these were combined together to create averaged MAPI and MAI PDOS curves and used only the ground-state orbitals, which excludes final-state orbital relaxation effects present in the experimental XPS process. These calculations used the PBE functional with fully or scalar-relativistic PBE ultrasoft pseudopotentials (USPP), respectively, with a wave function cutoff of 55 Ry and a charge density cutoff of 600 Ry. As with the CP2K results, Gaussian convolution was employed to generate smooth curves from discrete intensities with a broadening of σ = 0.2 eV. The curves without SOC were shifted to match non-SOC results with the experimental spectra, and the same shift applied to the respective SOC curves, resulting in a shift of −4.23 eV for MAPI and −3.06 eV for MAI. The SOC curves were also normalized to the same integral as the non-SOC curves to allow a more direct comparison of the effect of SOC on peak position and broadening.

After running the MAI simulation for 50 ps, we noted there was a slight typo in the b parameter resulting in an erroneous starting value of 15.519 Å, a relative error of 0.9%, causing an *a*/*b* ratio different from unity, which persisted throughout the simulation because of the isotropic NPT barostat. To demonstrate if this had any effect on the results presented here, we redid the last 10 ps of simulation with the corrected a = b values, and analysis of the *g*(*r*) shows nearly identical results indicating that the cell difference has negligible effects on the dynamics and structure of the systems being studied here.

### Experimental Details

In this study, we include experimental data from two different measurement campaigns, targeting different aspects of the electronic structure of MAPI and MAI. The occupied energy levels are probed via XPS with a photon energy primarily sensitive to heavy elements, and the unoccupied energy levels can be probed with nitrogen K-edge XA spectroscopy, revealing the local electronic structure at the nitrogen sites with particular sensitivity to the N p character.

#### Valence XPS

Valence band measurements were carried out on a single-crystal sample of MAPbI_3_ and a thin film of MAI on a TiO_2_ substrate at the FlexPES beamline at MAX IV synchrotron facility, Lund, Sweden. The MAPbI_3_ single crystal was prepared according to a previously reported procedure and cleaved in vacuum prior to measurement.^44^ Methylammonium iodide thin films were deposited on fluorine-doped tin oxide (FTO)/TiO_2_ substrates prepared in the following way: the FTO-coated glass was cleaned following a three-step method, placing the substrates in an ultrasonic bath filled first with RBS 50 detergent + water, second with ethanol and finally with acetone. The substrates were dried and subsequently placed in a UV-ozone cleaner for 10 min. After that, a compact layer of TiO_2_ was deposited using spray pyrolysis. A solution consisting of ethanol, acetyl acetone, and titanium diisopropoxide (30% in isopropanol) (90:4:6 by volume) was used. The substrates were heated on a hotplate and kept at 450 °C for 15 min before the spray deposition. 10 mL of solution was used to cover 200 cm^2^ of the substrate and air at 1 bar was employed as the carrier gas, obtaining a compact layer of anatase with an approximate thickness of 20–30 nm. 0.8 M MAI solution in *N*,*N*-dimethylformamide (DMF) was prepared by adding 129.77 mg of methylammonium iodide (Sigma-Aldrich 98%) per 1 mL of DMF (Sigma-Aldrich, ACS reagent, ≥99.8%) in a glove box and without any further treatment. The solution was stirred at room temperature and filtered through a 0.22 μm PTTA filter right before the deposition. The MAI solution was spin-coated in a glove box with an inert atmosphere. 75 μL of precursor solution was spread over each substrate (1.5 × 2.5 cm) and spin-coated using a one-step program using a rotation speed of 3500 rpm with an acceleration of 3500 rpm s^–1^ for 20 s. Directly after spin-coating, the films were placed in a hotplate at 70 °C for 30 min for annealing. After that, the samples were packed under N_2_ atmosphere and transported to MAX IV to be measured.

A photon energy of 390 eV was chosen using the plane grating monochromator and an exit slit of 8 μm at the FlexPES beamline. Photoelectron spectra were measured in a binding energy region of 56 to −1 eV using a Scienta-Omictron DA30-L(W) analyzer in normal emission from the samples with a pass energy of 100 eV. This region includes the I 4d core level and the Ti 3p core level of the TiO_2_ substrate. To remove contributions of the TiO_2_ substrate from the valence band spectrum of MAI, the spectra obtained from a blank TiO_2_ film and the MAI on TiO_2_ were normalized to the Ti 3p intensity and the TiO_2_ signal was then subtracted from the measured spectrum of MAI on TiO_2_. To compare the valence band spectra between MAPbI_3_ and MAI, the spectra were energy calibrated to the position of the I 4d core level and normalized to its intensity.

#### Nitrogen K-Edge XA Spectroscopy

For evaluation of the theoretical results on the PDOS of the unoccupied levels and of the simulated nitrogen K-edge XA spectra, we compare to experimental total electron yield (TEY) N 1s XA spectra, where the raw data is taken from Figure S7 in the supplementary material of our previous study of electronic couplings in MAPI.^22^ The experimental N 1s XA spectra, reproduced in the current study, are the result of background subtraction. For MAPI, the reference XA spectrum for PbI_2_, which lacks nitrogen, was subtracted to remove the Pb N_5_ signal which overlaps with the nitrogen data. This was followed by the subtraction of a linear background for both MAPI and MAI. The MAPI sample was a single crystal cleaved in ultrahigh vacuum (UHV) just prior to the measurement, whereas powder samples were used for MAI and PbI_2_. These bulk-sensitive measurements were performed at the LowDosePES^45^ endstation at the BESSY II synchrotron in pseudo single bunch mode using soft X-ray beamline PM4. Further details on the experimental conditions can be found in an Auger spectroscopy study on MAPI,^22^ in which the measured XA spectra were originally reported. The experimental N 1s XA spectrum for MAPI has also been used in an investigation of halide substitution in methylammonium lead halide perovskites.^20^

## Results and Discussion

**Figure 1:**
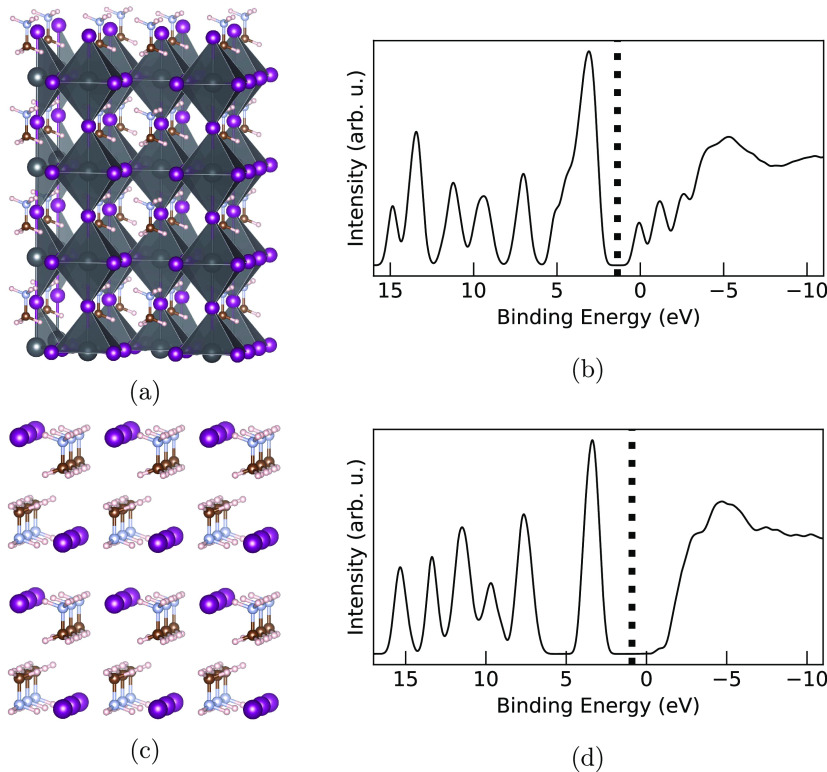
Starting supercell geometries and AIMD-sampled total electronic density of states of the Kohn–Sham orbitals for (a, b) MAPI and (c, d) MAI. In (a) and (c), the carbon, nitrogen, hydrogen, lead, and iodine atoms are brown, blue, white, gray, and purple, respectively. In (b) and (d), the center of the average band gap for the sampled geometries, defined as the difference between the highest occupied molecular orbital (HOMO) and lowest unoccupied molecular orbital (LUMO) orbital energies, after applying the shift to experimental data discussed in the computational details, is shown by the dashed line.

Through comparison of the electronic structure of the MAPI and MAI materials, we aim to investigate the effect of differences in the inorganic components, in particular the chemical bonding in the inorganic framework in MAPI and how the lead ions affect the interaction between the organic MA^+^ cations and the iodide ions. Figure 1 shows the starting crystal geometries for the MAPI and MAI systems as well as the total density of states of occupied and unoccupied Kohn–Sham orbitals from electronic ground-state DFT calculations at the geometries sampled from their respective AIMD simulations.

In Figure 1a, the structure of MAPI is shown to be composed of a PbI_3_^–^ lattice, ordered in a corner-sharing PbI_6_ octahedral structure, with MA^+^ counterions residing in cavities between these octahedra. The MA^+^ ions are able to rotate relatively freely in their cages at room temperature. In contrast, the MA^+^ ions in MAI form staggered layers with iodine atoms in between, as shown in Figure 1c, and these ions have been seen experimentally to remain vertical along the *c* axis with disordered methyl orientation.^24^Figure 1b,d shows the calculated electronic density of states for MAPI and MAI, respectively, for the occupied and unoccupied orbitals around the Fermi level. These curves are averaged over calculations performed on the five sampled snapshots for each material and are in good agreement with previous results.^11,13^ The main features of the occupied levels are similar in the two materials, with the striking difference being the shoulder in the valence band peak of MAPI around 4.7 to 5.8 eV compared to MAI. Analogously, the unoccupied levels give a broad feature around −3.5 to −7.5 eV in both MAPI and MAI, but there are clear differences in the region close to the conduction band minima where MAPI exhibits three distinct peaks from 1 to −3 eV compared to MAI which shows smoothly increasing density of states with small shoulders at −0.7 and −3.1 eV. Further analysis of the similarities and differences in the electronic density of states of MAPI and MAI requires decomposition into assigned contributions, which is presented below after the discussion of the purely structural degrees of freedom.

### Structure and Hydrogen Bonding in MAPI and MAI

An important aspect of the combination of AIMD simulations and sampled PDOS calculations is that it allows us to establish a link between the structural and electronic properties of MAPI and MAI, with a particular focus on the interactions between the organic cations and the inorganic ions. For this purpose, a structural analysis is performed on the basis of *g*(*r*) between atoms in the organic and inorganic components.

**Figure 2:**
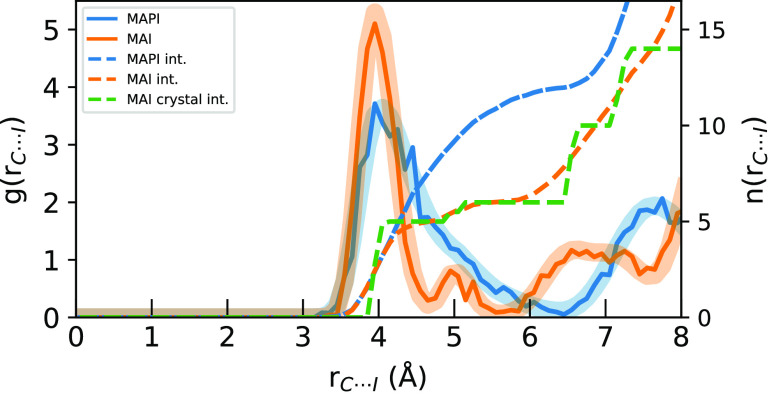
Radial distribution functions (solid) and their *n*_*I*_ integrals (dashed) of C···I distances for the sampled geometries (solid) compared to the full simulation (transparent) for MAPI (blue) and MAI (orange) AIMD simulations. The integral of the starting MAI crystal structure is shown in green.

In Figure 2, the C···I *g*(*r*) are shown to have broad peaks in the first coordination shell, clearly separated from the second shell at 6.5 Å for MAPI and 5.7 Å for MAI as a consequence of their crystal structures. The first peak is asymmetric, and in the case of MAI shows some substructure, both aspects associated with noncentrosymmetric coordination in the inorganic cages. In MAPI, the MA^+^ ions are confined to cuboctahedral cages where 12 iodide ions reside at the edges of the cube. The *n*_*I*_ integral of the AIMD-sampled MAPI *g*(*r*) (blue dashed) shows that the first peak integrates to 12 at 6.5 Å, corresponding to the *g*(*r*) minimum between the first and second shells. In MAI, the MA^+^ ions are clearly asymmetrically positioned in distorted octahedra with one C···I distance distinctly longer than the others, manifesting as a separate *g*(*r*) substructure at 5 Å. This is readily understood from the layered crystal structure of MAI seen in Figure 1c. The integrals of the AIMD-sampled MAI *g*(*r*) (orange dashed) and the starting crystal structure (green dashed) are shown for comparison. Figure 2 shows that this first peak in the AIMD-sampled *g*(*r*) for C···I in MAI integrates to 5 at 4.7 Å, with the next iodine “across” the C–N bond being notably farther than the others and completing the octahedral coordination of 6 at 5.6 Å. The close match between the integrals of the AIMD samples and the experimentally determined crystal structure demonstrates that the ionic framework of MAI does not deform significantly during the AIMD simulation. This comparison is feasible to do for MAI because the fluctuations in the orientation of the MA^+^ ions are relatively small during the simulation; for MAPI, which displays more free rotation of the MA^+^ ions, a fixed starting structure will be a significantly worse representation of the AIMD-sampled geometries.

**Figure 3:**
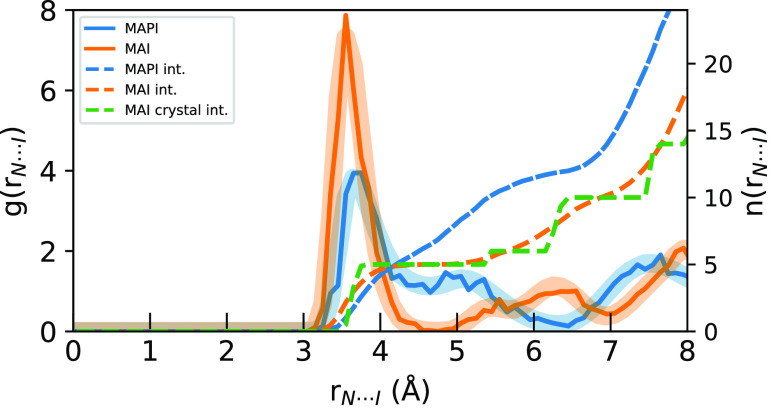
Radial distribution functions (solid) and *n*_*I*_ their integrals (dashed) of N···I distances for the sampled geometries (solid) compared to the full simulation (transparent) for MAPI (blue) and MAI (orange) AIMD simulations. The integral of the starting MAI crystal structure is shown in green.

In Figure 3, the corresponding N···I *g*(*r*) are compared. In comparison to the C···I *g*(*r*) in Figure 2, we note that the asymmetry in coordination to the MAPI cage iodide ions is even more pronounced and contributions from the cage separate into two distinct features. For MAPI, there is a sharp N···I peak at 3.7 Å and a broad feature around 5 Å, each with approximately equal *n*_*I*_ integrals summing to 12 at 6.5 Å, thereby accounting for the 12 neighboring iodide ions residing at the vertices of each cuboctahedral cage. In MAI, a distinct N···I peak integrating to 5 occurs at an even shorter distance of 3.5 Å and is completely separated from the sixth N···I distance at ∼5.5 Å which completes the distorted octahedral cage of 6 iodine atoms, which then merges with contributions from the second coordination shell. The radii of the peak maxima for the C···I and N···I *g*(*r*) in Figures 2 and 3 and the asymmetries and substructures in the first coordination shells show that the MA^+^ ions are primarily interacting with iodide ions through the charged ammonium group, resulting in shorter distances and greater asymmetries for N···I than for C···I.

**Figure 4:**
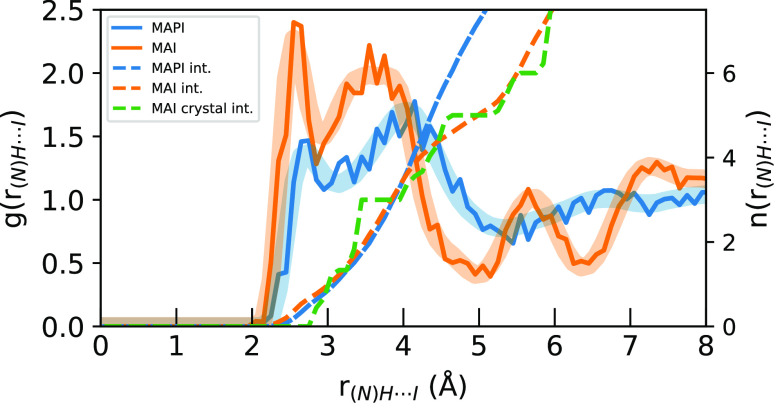
Radial distribution functions (solid) and their *n*_*I*_ integrals (dashed) of (N)H···I distances for the sampled geometries (solid) compared to the full simulation (transparent) for MAPI (blue) and MAI (orange) AIMD simulations. The integral of the starting MAI crystal structure is shown in green.

**Figure 5:**
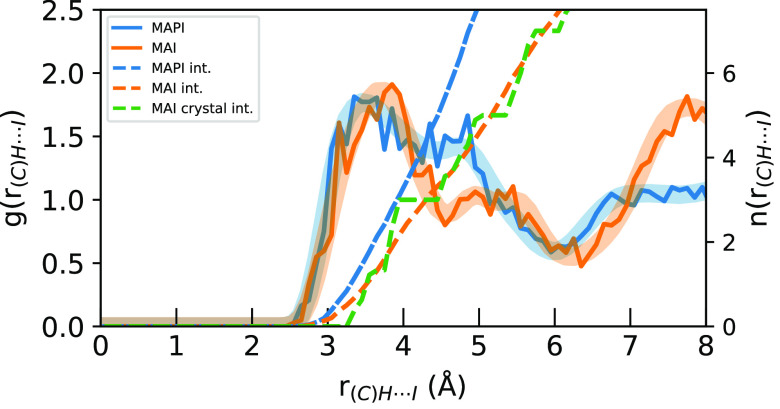
Radial distribution functions (solid) and their *n*_*I*_ integrals (dashed) of (C)H···I distances for the sampled geometries (solid) compared to the full simulation (transparent) for MAPI (blue) and MAI (orange) AIMD simulations. The integral of the starting MAI crystal structure is shown in green.

Figures 4 and 5 show the H···I *g*(*r*) for the hydrogen atoms attached to the nitrogen and carbon atoms, respectively, in MAPI and MAI. In Figure 4, it is apparent that the first (N)H···I peak in MAI has a slightly shorter distance and nearly twice the intensity as the corresponding peak in MAPI, indicating stronger H-bonding in MAI than in MAPI. However, we can see that the integrated (N)H···I *g*(*r*) for the two materials are similar in the short range below 4 Å, increasing later due to the fact that MAPI has nearly 1.5× the iodine number density of MAI. In MAI, the closest 5 iodine atoms in the distorted octahedron are accounted for in the first two peaks, seen as the *n*_*I*_ integral reaches 5 around 5 Å, and the 6th atom is accounted for by the 3rd peak at 5.7 Å. In MAPI, the contributions are less clear due to less constrained MA^+^ rotation.

In Figure 5, the (C)H···I *g*(*r*) are similar to those of the (N)H···I ones, but with some key differences. In particular, it is again clear from Figure 1 that the MAI carbon atoms are in general further away from the iodine atoms than the nitrogen atoms due to the crystal structure of the material, while in MAPI they are more similar due to MA^+^ rotation. The distances for both materials are still longer in the carbon case because the closer (N)H distances means that the corresponding (C)H must be further away across the N–C bond.

Finally, we note that in all cases, the *g*(*r*) from the limited sampling over five configurations (used in the PDOS analysis below) closely follows the *g*(*r*) from the full trajectory, indicating that the sampled configurations are representative of both materials and can allow us to perform a relevant analysis of the electronic structure.

### Analysis of XPS Measurements and PDOS Calculations

**Figure 6:**
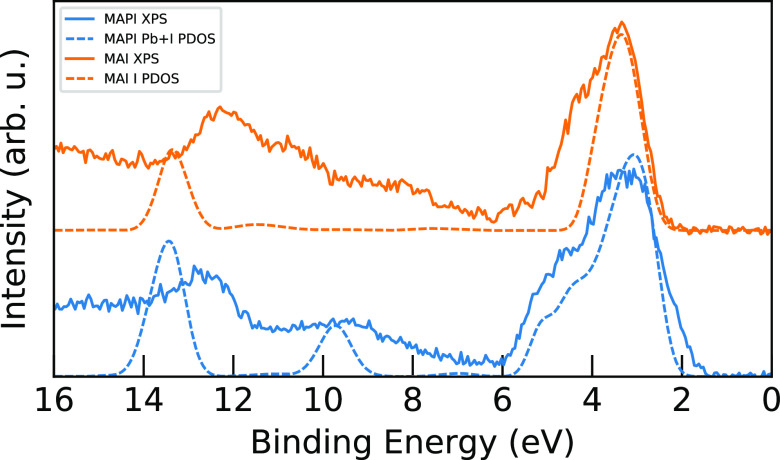
Comparison of experimental XPS data measured with a photon energy of 390 eV (solid) and calculated I + Pb PDOS (dashed) results for MAPI (blue) and I PDOS for MAI (orange). The calculated PDOS have been shifted by −2.68 and −2.49 eV for MAPI and MAI, respectively, to align with the experimental values of the valence band peaks between 2.5 and 4 eV.

Following this overall comparison of geometric differences, the experimental valence XPS data for MAPI and MAI is analyzed in terms of theoretical PDOS in Figure 6, which compares calculated occupied PDOS of lead and iodine to experimental XPS data for MAPI and MAI, measured with a photon energy of 390 eV.

In these measurements, sensitivity to photon energy in the ionization cross sections of different elements and levels can be used to enhance certain features.^12^ From previous studies of the photon energy dependence of the XPS spectrum,^12^ we expect the nitrogen and carbon levels to have a small, but not negligible contribution, at 390 eV. These spectra are more sensitive to the heavier Pb and I atoms, thus only the PDOS of these elements are included in the comparison here. Consequently, the calculated PDOS plots for both materials lack intensity in large regions: from 5.8 to 12.5 eV for MAPI, with the exception of one peak at 9.7 eV from the Pb 6s, and from 4.7 to 12.5 eV for MAI, due to the neglected carbon and nitrogen contributions.

It can be seen that the calculated PDOS curves match the main features in the experimental XPS spectra, with primarily a slight difference in the dispersion of the peaks, leading to the calculated PDOS curves being more stretched than the XPS spectra. As seen above, when comparing the two materials, we note that the valence band in MAPI at 1.3 to 5.8 eV is broader and more asymmetric than in MAI. Since this binding energy region is dominated by I 5p levels,^12^ the phenomenon is related to hybridization with lead levels, which will be analyzed in detail below by angular decomposition of the I 5p and Pb PDOS. Here we note that the PDOS is expected to be different since MAPI has a PbI_6_ network with hybridized orbitals and weaker contribution from MA^+^, whereas the MAI has valence states due to I 5p orbitals hybridized with the lowest unoccupied molecular orbital of isolated MA^+^ ions. However, the PDOS from the model of the MAI material does not capture the shoulder at 4.5 eV, which as seen below is in a region lacking features of organic character. Unlike MAPI, the MAI sample was not a single crystal cleaved under vacuum and therefore this shoulder could be related to surface impurities or defects.

**Figure 7:**
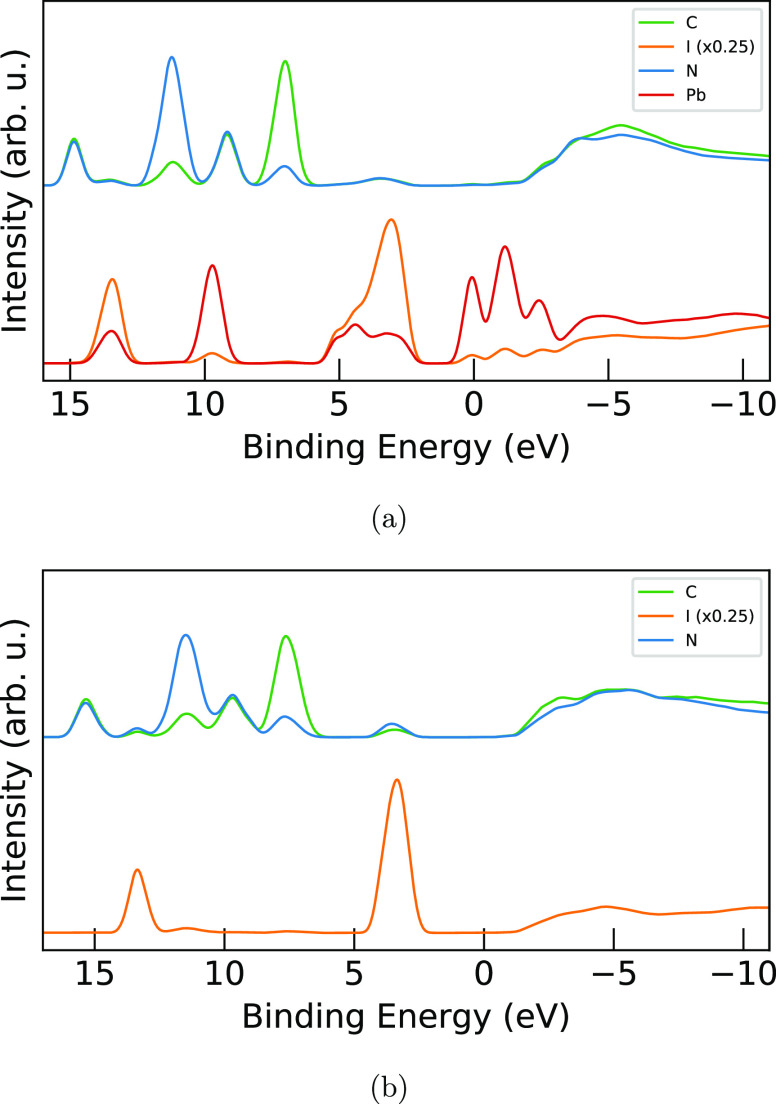
Comparison of element-specific PDOS for (a) MAPI and (b) MAI geometries from the AIMD simulations. Carbon is shown in green, iodine in orange, nitrogen in blue, and lead in red. The notable qualitative differences between these systems seen in Figure 1, specifically around the valence and conduction bands, are here seen to be primarily a result of the hybridization of the iodine with the lead levels, with an additional small contribution of MA^+^ hybridization due to hydrogen bonding.

The experimental MAPI and MAI peaks at 12.6 and 12.2 eV, associated with I 5s levels, correspond to the calculated peaks at around 13.4 and 13.3 eV, respectively. The calculated MA^+^ levels are discussed below in Figure 7, but we are not able to resolve them individually in the experimental spectra, only broad features in the region 7–11 eV (though we bring the reader’s attention to previous measurements using X-ray emission spectroscopy for MAPI^21^ and MAI^26^). Despite this, there is clear general agreement between the calculated and experimental spectra presented here, which allows a more in-depth analysis of the calculated data.

We note here that both the calculated and experimental spectra presented here lack the low-intensity valence band maximum features seen in some previous studies of lead halide perovskites.^13,46^ The calculations used to demonstrate this were performed using *k*-point sampling, while our supercell structures are constrained to the Γ point. Furthermore, we have used dynamic sampling which may smooth out these low-intensity peaks. Experimentally, these were shown using sensitive UPS spectra, while the XPS spectra presented here have a lower signal-to-noise ratio and are thus not sensitive enough to see this low-intensity tail.

### Analysis of Band Gap Differences in MAPI and MAI

As can be seen clearly in the PDOS curves in Figure 1, the electronic structures of these two materials are overall quite similar but have a number of distinct differences. In particular, the valence band of MAPI from 5.8 to 1.3 eV is much broader and more asymmetric than that of MAI, and the conduction band has been pulled down significantly and contains a few sharp features due to PbI_6_ hybridized orbitals signifying differences in chemical bonding. These factors contribute to a large decrease in the experimental band gap of MAPI (1.55 eV) relative to MAI (3.12 eV),^13,47^ also reflected in our calculated PBE band gaps of 1.85 and 3.60 eV, respectively. The calculated band gaps reported here are somewhat higher than that of previous studies which generally report a value around 1.6 eV for optimized MAPI with the PBE functional, though these also employ *k*-point sampling while we do not.^13,15,48^ However, we note that structural fluctuations and cationic orientation can cause large variation in the calculated band gap, increasing it even up to 1.8 eV in similar PBE Γ-point calculations sampled from AIMD.^49,50^ We also note that we have an overestimation of the band gap for MAI, though this is reduced by about 0.45 eV by the addition of spin–orbit coupling.

Decomposition of the total density of states into contributions from C, N, I, and Pb in the case of MAPI (seen in Figure 7a) shows that the narrowing of the band gap mainly comes from the influence of lead hybridizing with the iodine levels in the conduction band, and not significantly involving the MA^+^ levels.^51^ We see that lead contributed to the full width of the valence band feature at 5.8 to 1.3 eV and dominates at the bottom of the conduction band. At 13.3/4 eV, we see that the I 5s level is hybridized with Pb 6s and 6p. Reversely at 9.7 eV, the Pb 6s level has an admixture of I 5p. For MAI, however, the I 5p and I 5s levels exhibit sharp features, and in particular at 11.4 eV features of small iodide PDOS intensity are correlated with high nitrogen PDOS.

Examination of the *g*(*r*) of (N)H···I can provide some insight into the differences in the PDOS between MAPI and MAI. As noted above, the first peak for (N)H···I in MAI occurs at a slightly shorter distance and has nearly twice the intensity in comparison to the same peak for MAPI, indicating that there is stronger (N)H···I interaction (hydrogen bonding) in the MAI system. Correspondingly, in the valence band maximum, the MAI MA^+^ levels are more intense and coupled with the iodine levels in a narrow-energy region, while the MAPI MA^+^ levels are more dispersed by further influence of the lead (see the C/N features at 3.5 eV in the valence peak in Figure 7b, compared to the corresponding broad features in Figure 7a). In contrast, the main C PDOS and N PDOS are narrower in MAPI than in MAI. As explored in previous nitrogen K-edge X-ray emission and Auger spectroscopy studies,^21,22,26^ hydrogen bonding gives a weak mixing of N PDOS with the I 5p PDOS in the upper valence band but in the conduction band the σ bonding in sp^3^ hybridized carbon and nitrogen in the MA^+^ ion gives a broad feature in the unoccupied PDOS starting at −2 eV with a peak around −5 eV.^21,22^ This influence of hydrogen bonding between MA^+^ and I^–^ ions is most prominent at the I 5p levels in the nitrogen PDOS and at 13.3–4 eV in both materials, but is also responsible for the weak features in the I PDOS at 11.4 and 7.5 eV in MAI. The influence of differences in interactions on the unoccupied level of the MA^+^ ions are mainly seen in the slight redistribution of nitrogen and carbon character, associated with the stronger hydrogen bonding in MAI in comparison to MAPI. We interpret this reduction of nitrogen character at the bottom of the conduction band and shift further up in the conduction band to be due to stronger hydrogen bonding in MAI. It is a signature of the hydrogen-bonding interaction, complementary to the changes in the occupied levels, as observed in, e.g., the N 1s XA spectrum of ammonia upon hydration.^52^

**Figure 8:**
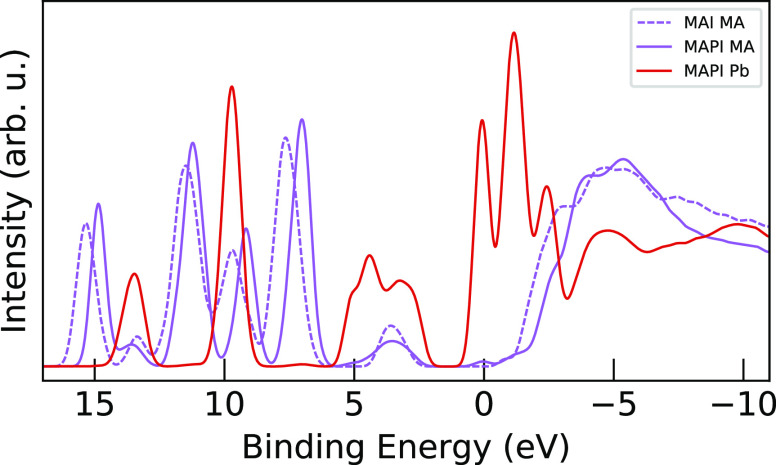
AIMD-sampled PDOS for MA^+^ atoms in MAI (dashed purple) and MAPI (solid purple) presented together with the Pb PDOS in MAPI (red) levels to show its effect on the MA^+^ levels: pulling down the valence band and interacting in the conduction band.

**Figure 9:**
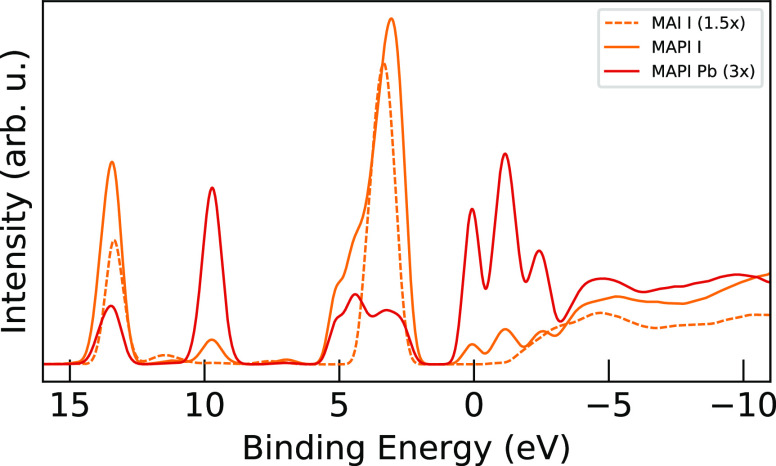
AIMD-sampled PDOS for iodine in MAI (dashed orange) and MAPI (solid orange) presented together with the Pb PDOS in MAPI (red) to show its effect on the iodine levels.

**Figure 10:**
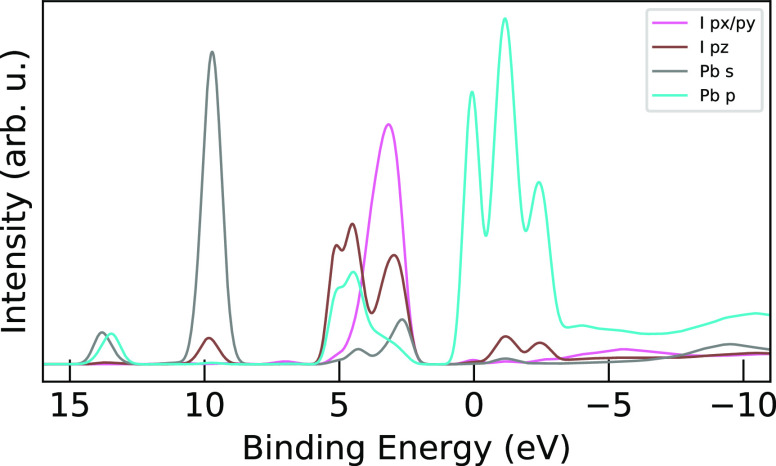
In MAPI, the iodine p PDOS split into the p_*x*/*y*_ (pink) and p_*z*_ (brown) components for the vertically bonded lattice iodine atoms, and the lead levels shown for s (gray) and p (light blue) orbitals.

The influence of the Pb^2+^ ions and the difference in crystal structures on the MA^+^ levels is shown in Figure 8. There is a general shift seen between the two materials of approximately 1 eV in MAPI relative to MAI, which we suggest is due to the MA^+^ ion experiencing a different electrostatic field from the different crystal environments. This then highlights the valence band region (from 2.1 to 5.8 eV) where the MA^+^ state maximum has not shifted, but rather has spread in MAPI due to hybridization with the inorganic lattice through the iodine and lead. Similar behavior is seen around 13.5 eV where the MAPI peak has shifted oppositely to a slightly higher binding energy toward the Pb 6s/p peak. We can also see a clear increase in the MA^+^ levels in the conduction band shoulder (from 1 to −1.8 eV) before the main rising edge in MAPI, matching the spikes of lead levels in that region. These effects are smaller compared to the changes seen in the iodine levels in Figure 9. The Pb–I bonding is discussed in more detail below in Figure 10 with an angular decomposition of the I p contribution to the density of states.

In Figure 9, the difference in iodine levels between MAI and MAPI is examined in detail along with the lead levels. The shoulder in the I PDOS of MAPI in the valence band around 4–5.5 eV correlates well with the appearance of the lead levels, representing an I 5p to Pb 6p interaction.^12^ Similar behavior is seen in the shift of the peak around 14 eV, and even more clearly with the MAPI peak at 9.7 eV moving exactly to the position of the lead levels there, in contrast to the MAI iodine levels appearing more at 11.5 eV. Also the increase of iodine levels in the conduction band of MAPI from 1 to −3 eV matches almost exactly the shape of the lead levels there.

In Figure 10, we show the difference in the I PDOS of the Cartesian components of the I p orbitals (p_*x*_ = p_*y*_, and p_*z*_) for the iodine atoms vertically bonded to lead (i.e., Pb–I–Pb along the *z* axis) and how they compare to the lead 6s and 6p levels. We can see regarding the asymmetric shoulder in the valence band from 4 to 6 eV that the vertically bonded iodine atoms see this shoulder appear essentially as a result of the p_*z*_ components only, as a result of σ chemical bonds with the lead. This is also seen in the peak at 9.7 eV. Close inspection of the three prominent conduction band peaks from 1 to −3 eV shows that p_*x*_ and p_*y*_, representing π interactions, and the p_*z*_ σ bond all contribute equally to the first peak, while the second and third peaks are predominantly from I p_*z*_ sigma bonds, in agreement with previous research on the conduction band of bromide HOIPs.^27^ Moreover, the I p_*z*_ levels match reasonably closely the lead levels in the valence band edge because of the σ character, while the p_*x*_ and p_*y*_ levels with their out-of-plane π character do not.

In summary, Figures 8–10 show the environment around the MA^+^ ions in the two materials. For MAI, the distance between I ions is relatively larger than in MAPI, and there is stronger MA^+^–I^–^ interaction. Hybridization of PbI_6_ octahedra gives rise to distinct features in the valence band maximum with the asymmetric shoulder near 5 eV and even broadening toward the Fermi level, as well as dominant Pb–I features in the conduction band.

### Analysis of Nitrogen K-Edge XA Spectra

**Figure 11:**
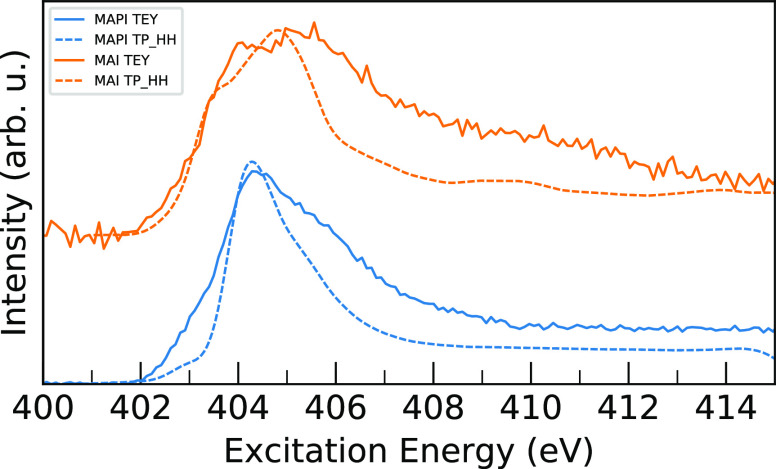
Comparison of calculated TP_HH N 1s XA spectra (dashed) and experimental TEY N 1s XA spectra (solid) for MAPI (blue) and MAI (orange). Experimental data are taken from our previous study of electronic couplings in MAPI,^22^ following a data treatment described in the Experimental Details section.

Figure 11 compares calculated TP_HH N 1s XA spectra with experimental TEY N 1s XA spectra. The calculated spectra have both been shifted by a constant shift to align the main peak for MAPI with experiment; the same shift was used with MAI to preserve the relative shift of the two materials, which agrees quite well between theory and experiment. Similar to the valence band results, the calculated N 1s XA spectrum is more narrow than the experimental one, a fact that has been previously observed in ammonium calculations and previous work on this MAPI system under the transition potential approximation.^20,53,54^ Nonetheless, these results show generally quite good agreement in spectral features between the experimental and calculated spectra, validating the structural models used here. In particular, the calculated N 1s XA spectrum for MAPI reproduces the single peak around 404 eV and the calculated N 1s XA spectrum for MAI reproduces the double-peak character from 404 to 406 eV. As the onset of the spectra for MAI is well reproduced in the calculations, the shoulder at 403 eV for MAPI is strongly underestimated in the calculations, as previously observed for aqueous ammonium^53^ at the same level of theory. The post-edge region is poorly described in both models as observed previously for the N 1s TP_HH method, a shortcoming requiring higher-level quantum chemistry to resolve.^55,56^

### Addition of Exact Exchange and Spin–Orbit Coupling

**Figure 12:**
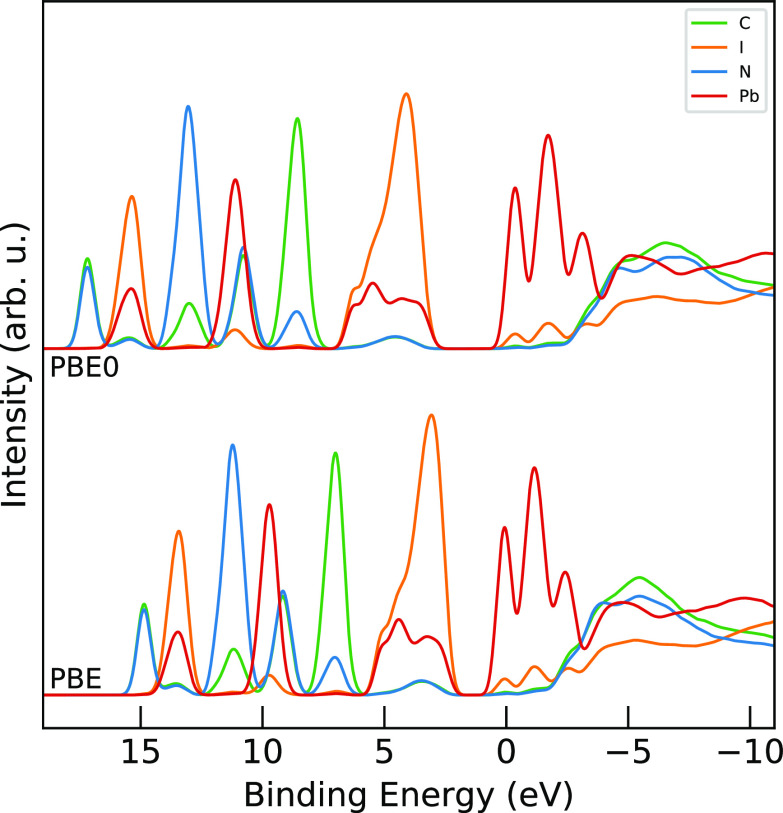
Comparison of PDOS for MAPI with PBE (bottom) and hybrid PBE0 (top) functionals split along elements: carbon in green, iodine in orange, nitrogen in blue, and lead in red.

We also investigated the effects of inclusion of exact exchange (through the hybrid functional PBE0^57^) and spin–orbit coupling (SOC) on the PDOS curves. The PDOS of MAPI, split along element components for PBE and its hybrid cousin PBE0, are shown in Figure 12. The PBE0 curves have the same -2.68 eV shift as the previous PBE curves. Though the PBE0 curves are more spread out than the PBE ones, the relative peak structure is essentially the same between the two functionals. In particular, the shapes of the valence band maximum and conduction band minimum are nearly identical. However, due to the stretching in PBE0, the band gap of MAPI with PBE0 is significantly too large, which is an established fact related to the fortuitous error cancellation found with PBE.^15,48^ We also stress that the increased dispersion of the occupied PDOS for the PBE0 functional results in worse agreement with the experimental XPS data seen in Figure 6.

**Figure 13:**
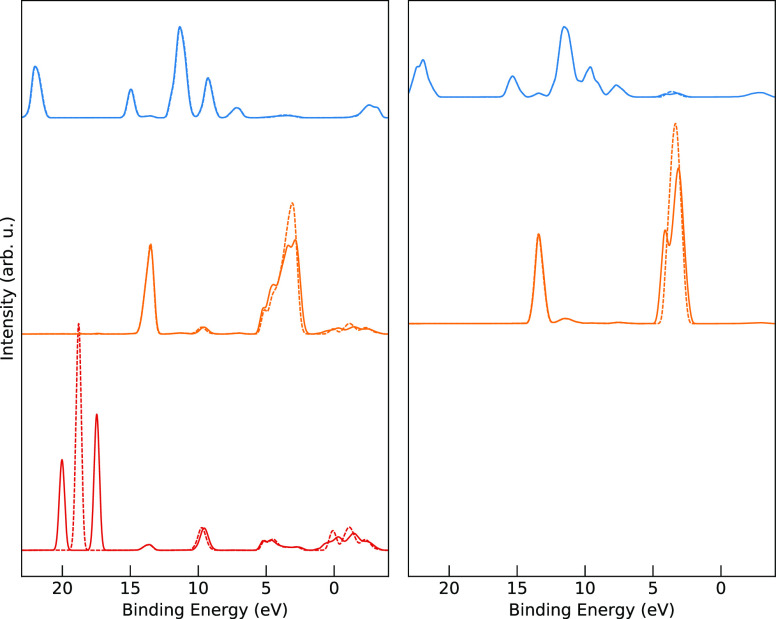
Comparison of nitrogen (blue) and iodine (orange) PDOS with (solid) and without (dashed) the effects of spin–orbit coupling for MAPI (left) and MAI (right).

Further PDOS calculations on MAPI using the PBE functional with and without SOC, performed using Quantum ESPRESSO, are presented in Figure 13 (left) with solid and dashed lines, respectively, and similarly for MAI on the right-hand side. In both materials, the PDOS for nitrogen (blue, top) shows little influence of SOC, as expected because it is a light element. On the other hand, the PDOS of the heavier elements of iodine (orange, center) and lead (red, bottom) show distinct effects of SOC. For iodine, the SOC mainly causes a broadening of the valence band peak with a smaller effect on the conduction band. Conversely, for lead in MAPI, in addition to the large splitting of the 4d peak around −18 eV binding energy, the inclusion of SOC causes only a small effect in the valence band but a clear broadening of the conduction band minimum region. This results in a decreased band gap for both materials, with a more pronounced reduction in MAPI due to the changes in both iodine and lead which is lacking in MAI.

## Conclusions

In this work, we have compared experimental XPS and N 1s XA spectra for the perovskite MAPbI_3_ (MAPI) and its precursor MAI with calculated PDOS and theoretical N 1s XA spectra coming from snapshots of a molecular dynamics simulation. The calculated models agree very well with the experimental data for both XPS and XA spectroscopy. Close examination of the electronic structures of the two materials reveals that they are overall similar in terms of number of peaks and positions, especially with regard to occupied orbitals near the valence band maximum, but there are two clear differences we focus on here: valence band peak asymmetry in MAPI relative to MAI and the relative decrease in band gap in MAPI. By partitioning the PDOS spectra according to atom type, it can be seen that both of these differences can be clearly explained by lead hybridization, mainly with iodine but also with the MA^+^ levels. This can also be rationalized with the N(H)···I *g*(*r*) differences between the two materials, which indicates that there is a much stronger N(H)···I interaction in MAI than MAPI. However, as shown in a previous study of lead bromide perovskites,^27^ the interaction between MA^+^ and I^–^ influences the relative energy positions of σ*(Pb–I) and π*(Pb–I) levels in the conduction band, suggesting that these interactions could then in theory be fine-tuned to improve perovskite solar cell function.

## Notes

Data sets generated during the current study are available from the corresponding authors upon reasonable request.

## References

[ref1] Hou X.; Wang X.; Mi W.; Du Z. Prediction on Electronic Structure of CH_3_NH_3_PbI_3_/Fe_3_O_4_ Interfaces. Solid State Commun. 2018, 269, 90–95. 10.1016/j.ssc.2017.10.019.

[ref2] Lindblad R.; Bi D.; Park B.-w.; Oscarsson J.; Gorgoi M.; Siegbahn H.; Odelius M.; Johansson E. M. J.; Rensmo H. Electronic Structure of TiO_2_/CH_3_NH_3_PbI_3_ Perovskite Solar Cell Interfaces. J. Phys. Chem. Lett. 2014, 5, 648–653. 10.1021/jz402749f. 26270831

[ref3] Kot M.; Wojciechowski K.; Snaith H.; Schmeißer D. Evidence of Nitrogen Contribution to the Electronic Structure of the CH_3_NH_3_PbI_3_ Perovskite. Chem. – Eur. J. 2018, 24, 3539–3544. 10.1002/chem.201705144. 29359824

[ref4] Kojima A.; Teshima K.; Shirai Y.; Miyasaka T. Organometal Halide Perovskites as Visible-Light Sensitizers for Photovoltaic Cells. J. Am. Chem. Soc. 2009, 131, 6050–6051. 10.1021/ja809598r. 19366264

[ref5] Jeong J.; Kim M.; Seo J.; Lu H.; Ahlawat P.; Mishra A.; Yang Y.; Hope M. A.; Eickemeyer F. T.; Kim M.; et al. Pseudo-halide Anion Engineering for α-FAPbI_3_ Perovskite Solar Cells. Nature 2021, 592, 381–385. 10.1038/s41586-021-03406-5. 33820983

[ref6] National Renewable Energy Laboratory. Best Research-Cell Efficiencies. https://www.nrel.gov/pv/assets/pdfs/best-research-cell-efficiencies-rev220630.pdf (accessed October 27, 2022).

[ref7] Egger D. A.; Bera A.; Cahen D.; Hodes G.; Kirchartz T.; Kronik L.; Lovrincic R.; Rappe A. M.; Reichman D. R.; Yaffe O. What Remains Unexplained about the Properties of Halide Perovskites?. Adv. Mater. 2018, 30, 1800691 10.1002/adma.201800691. 29569287

[ref8] Senno M.; Tinte S. Mixed Formamidinium-Methylammonium Lead Iodide Perovskite from First-principles: Hydrogen-bonding Impact on the Electronic Properties. Phys. Chem. Chem. Phys. 2021, 23, 7376–7385. 10.1039/D0CP06713J. 33876097

[ref9] Ke X.; Yan J.; Zhang A.; Zhang B.; Chen Y. Optical Band Gap Transition from Direct to Indirect Induced by Organic Content of CH_3_NH_3_PbI_3_ Perovskite Films. Appl. Phys. Lett. 2015, 107, 091904 10.1063/1.4930070.

[ref10] Wang L.; Yuan G. D.; Duan R. F.; Huang F.; Wei T. B.; Liu Z. Q.; Wang J. X.; Li J. M. Tunable Bandgap in Hybrid Perovskite CH_3_NH_3_Pb(Br_3-y_X_y_) Single Crystals and Photodetector Applications. AIP Adv. 2016, 6, 045115 10.1063/1.4948312.

[ref11] Lindblad R.; Jena N. K.; Philippe B.; Oscarsson J.; Bi D.; Lindblad A.; Mandal S.; Pal B.; Sarma D. D.; Karis O.; et al. Electronic Structure of CH_3_NH_3_PbX_3_ Perovskites: Dependence on the Halide Moiety. J. Phys. Chem. C 2015, 119, 1818–1825. 10.1021/jp509460h.

[ref12] Philippe B.; Jacobsson T. J.; Correa-Baena J.-P.; Jena N. K.; Banerjee A.; Chakraborty S.; Cappel U. B.; Ahuja R.; Hagfeldt A.; Odelius M.; Rensmo H. Valence Level Character in a Mixed Perovskite Material and Determination of the Valence Band Maximum from Photoelectron Spectroscopy: Variation with Photon Energy. J. Phys. Chem. C 2017, 121, 26655–26666. 10.1021/acs.jpcc.7b08948.

[ref13] Endres J.; Egger D. A.; Kulbak M.; Kerner R. A.; Zhao L.; Silver S. H.; Hodes G.; Rand B. P.; Cahen D.; Kronik L.; Kahn A. Valence and Conduction Band Densities of States of Metal Halide Perovskites: A Combined Experimental-Theoretical Study. J. Phys. Chem. Lett. 2016, 7, 2722–2729. 10.1021/acs.jpclett.6b00946. 27364125 PMC4959026

[ref14] Even J.; Pedesseau L.; Jancu J.-M.; Katan C. Importance of Spin-Orbit Coupling in Hybrid Organic/Inorganic Perovskites for Photovoltaic Applications. J. Phys. Chem. Lett. 2013, 4, 2999–3005. 10.1021/jz401532q.

[ref15] Das T.; Di Liberto G.; Pacchioni G. Density Functional Theory Estimate of Halide Perovskite Band Gap: When Spin Orbit Coupling Helps. J. Phys. Chem. C 2022, 126, 2184–2198. 10.1021/acs.jpcc.1c09594.

[ref16] Umari P.; Mosconi E.; De Angelis F. Relativistic GW Calculations on CH_3_NH_3_PbI_3_ and CH_3_NH_3_SnI_3_ Perovskites for Solar Cell Applications. Sci. Rep. 2015, 4, 4467 10.1038/srep04467. PMC5394751 24667758

[ref17] Etienne T.; Mosconi E.; De Angelis F. Dynamical Origin of the Rashba Effect in Organohalide Lead Perovskites: A Key to Suppressed Carrier Recombination in Perovskite Solar Cells?. J. Phys. Chem. Lett. 2016, 7, 1638–1645. 10.1021/acs.jpclett.6b00564. 27062910

[ref18] Wang K.; Ecker B.; Gao Y. Angle-Resolved Photoemission Study on the Band Structure of Organic Single Crystals. Crystals 2020, 10, 773 10.3390/cryst10090773.

[ref19] Lee M.-I.; Barragán A.; Nair M. N.; Jacques V. L. R.; Le Bolloc’h D.; Fertey P.; Jemli K.; Lédée F.; Trippé-Allard G.; Deleporte E.; et al. First Determination of the Valence Band Dispersion of CH_3_NH_3_PbI_3_ Hybrid Organic-Inorganic Perovskite. J. Phys. D: Appl. Phys. 2017, 50, 26LT02 10.1088/1361-6463/aa71e7.

[ref20] Sterling C. M.; Kamal C.; Man G. J.; Nayak P. K.; Simonov K. A.; Svanström S.; García-Fernández A.; Huthwelker T.; Cappel U. B.; Butorin S. M.; et al. Sensitivity of Nitrogen K-Edge X-ray Absorption to Halide Substitution and Thermal Fluctuations in Methylammonium Lead-Halide Perovskites. J. Phys. Chem. C 2021, 125, 8360–8368. 10.1021/acs.jpcc.1c02017. PMC8162417 34084262

[ref21] Wilks R. G.; Erbing A.; Sadoughi G.; Starr D. E.; Handick E.; Meyer F.; Benkert A.; Iannuzzi M.; Hauschild D.; Yang W.; et al. Dynamic Effects and Hydrogen Bonding in Mixed-Halide Perovskite Solar Cell Absorbers. J. Phys. Chem. Lett. 2021, 12, 3885–3890. 10.1021/acs.jpclett.1c00745. 33856793

[ref22] Man G. J.; Sterling C. M.; Kamal C.; Simonov K. A.; Svanström S.; Acharya J.; Johansson F. O. L.; Giangrisostomi E.; Ovsyannikov R.; Huthwelker T.; et al. Electronic Coupling Between the Unoccupied States of the Organic and Inorganic Sublattices of Methylammonium Lead Iodide: A Hybrid Organic-Inorganic Perovskite Single Crystal. Phys. Rev. B 2021, 104, L041302 10.1103/PhysRevB.104.L041302.

[ref23] Ong K. P.; Wu S.; Nguyen T. H.; Singh D. J.; Fan Z.; Sullivan M. B.; Dang C. Multi Band Gap Electronic Structure in CH_3_NH_3_PbI_3_. Sci. Rep. 2019, 9, 2144 10.1038/s41598-018-38023-2. 30765739 PMC6376135

[ref24] Yamamuro O.; Matsuo T.; Suga H.; David W. I. F.; Ibberson R. M.; Leadbetter A. J. Neutron Diffraction and Calorimetric Studies of Methylammonium Iodide. Acta Crystallogr., Sect. B: Struct. Sci. 1992, 48, 329–336. 10.1107/S0108768192000260.

[ref25] Bukleski M.; Dimitrovska-Lazova S.; Aleksovska S. Vibrational Spectra of Methylammonium Iodide and Formamidinium Iodide in a Wide Temperature Range. Maced. J. Chem. Chem. Eng. 2019, 38, 237–252. 10.20450/mjcce.2019.1940.

[ref26] Kamal C.; Hauschild D.; Seitz L.; Steininger R.; Yang W.; Heske C.; Weinhardt L.; Odelius M. Coupling Methylammonium and Formamidinium Cations with Halide Anions: Hybrid Orbitals, Hydrogen Bonding, and the Role of Dynamics. J. Phys. Chem. C 2021, 125, 25917–25926. 10.1021/acs.jpcc.1c08932. PMC8634158 34868447

[ref27] Man G. J.; Kamal C.; Kalinko A.; Phuyal D.; Acharya J.; Mukherjee S.; Nayak P. K.; Rensmo H.; Odelius M.; Butorin S. M. A-site Cation Influence on the Conduction Band of Lead Bromide Perovskites. Nat. Commun. 2022, 13, 3839 10.1038/s41467-022-31416-y. 35787623 PMC9253039

[ref28] Hutter J.; Iannuzzi M.; Schiffmann F.; VandeVondele J. CP2K: Atomistic Simulations of Condensed Matter Systems. Wiley Interdiscip. Rev.: Comput. Mol. Sci. 2014, 4, 15–25. 10.1002/wcms.1159.

[ref29] Poglitsch A.; Weber D. Dynamic Disorder in Methylammoniumtrihalogenoplumbates (II) Observed by Millimeter-wave Spectroscopy. J. Chem. Phys. 1987, 87, 6373–6378. 10.1063/1.453467.

[ref30] Perdew J. P.; Burke K.; Ernzerhof M. Generalized Gradient Approximation Made Simple. Phys. Rev. Lett. 1996, 77, 3865–3868. 10.1103/PhysRevLett.77.3865. 10062328

[ref31] Grimme S.; Antony J.; Ehrlich S.; Krieg H. A Consistent and Accurate *ab initio* Parametrization of Density Functional Dispersion Correction (DFT-D) for the 94 Elements H-Pu. J. Chem. Phys. 2010, 132, 154104 10.1063/1.3382344. 20423165

[ref32] Grimme S.; Ehrlich S.; Goerigk L. Effect of the Damping Function in Dispersion Corrected Density Functional Theory. J. Comput. Chem. 2011, 32, 1456–1465. 10.1002/jcc.21759. 21370243

[ref33] Lippert G.; Hutter J.; Parrinello M. The Gaussian and Augmented-plane-wave Density Functional Method for ab initio Molecular Dynamics Simulations. Theor. Chem. Acc. 1999, 103, 124–140. 10.1007/s002140050523.

[ref34] Goedecker S.; Teter M.; Hutter J. Separable Dual-space Gaussian Pseudopotentials. Phys. Rev. B 1996, 54, 1703–1710. 10.1103/PhysRevB.54.1703. 9986014

[ref35] Krack M. Pseudopotentials for H to Kr Optimized for Gradient-corrected Exchange-correlation Functionals. Theor. Chem. Acc. 2005, 114, 145–152. 10.1007/s00214-005-0655-y.

[ref36] VandeVondele J.; Hutter J. Gaussian Basis Sets for Accurate Calculations on Molecular Systems in Gas and Condensed Phases. J. Chem. Phys. 2007, 127, 114105 10.1063/1.2770708. 17887826

[ref37] Nosé S. A Unified Formulation of the Constant Temperature Molecular Dynamics Methods. J. Chem. Phys. 1984, 81, 511–519. 10.1063/1.447334.

[ref38] Iannuzzi M.; Hutter J. Inner-shell Spectroscopy by the Gaussian and Augmented Plane Wave Method. Phys. Chem. Chem. Phys. 2007, 9, 1599–1610. 10.1039/b615522g. 17429553

[ref39] Clark T.; Chandrasekhar J.; Spitznagel G. W.; Schleyer P. V. R. Efficient Diffuse Function-augmented Basis Sets for Anion Calculations. III. The 3-21+G Basis Set for First-row Elements, Li-F. J. Comput. Chem. 1983, 4, 294–301. 10.1002/jcc.540040303.

[ref40] Krishnan R.; Binkley J. S.; Seeger R.; Pople J. A. Self-Consistent Molecular Orbital Methods. XX. A Basis Set for Correlated Wave Functions. J. Chem. Phys. 1980, 72, 650–654. 10.1063/1.438955.

[ref41] Perdew J. P. Density Functional Theory and the Band Gap Problem. Int. J. Quantum Chem. 1985, 28, 497–523. 10.1002/qua.560280846.

[ref42] Giannozzi P.; Baroni S.; Bonini N.; Calandra M.; Car R.; Cavazzoni C.; Ceresoli D.; Chiarotti G. L.; Cococcioni M.; Dabo I.; et al. Quantum ESPRESSO: a Modular and Open-source Software Project for Quantum Simulations of Materials. J. Phys.: Condens. Matter 2009, 21, 395502 10.1088/0953-8984/21/39/395502. 21832390

[ref43] Giannozzi P.; Andreussi O.; Brumme T.; Bunau O.; Buongiorno M.; Calandra M.; Car R.; Cavazzoni C.; Ceresoli D.; Cococcioni M.; et al. Advanced Capabilities for Materials Modelling with Quantum ESPRESSO. J. Phys.: Condens. Matter 2017, 29, 465901 10.1088/1361-648X/aa8f79. 29064822

[ref44] Fernandez A. G.; Svanström S.; Sterling C. M.; Gangan A.; Erbing A.; Kamal C.; Sloboda T.; Kammlander B.; Man G. J.; Rensmo H.; et al. Experimental and Theoretical Core Level and Valence Band Analysis of Clean Perovskite Single Crystal Surfaces. Small 2022, 18, 2106450 10.1002/smll.202106450. 35122466

[ref45] Giangrisostomi E.; Ovsyannikov R.; Sorgenfrei F.; Zhang T.; Lindblad A.; Sassa Y.; Cappel U. B.; Leitner T.; Mitzner R.; Svensson S.; et al. Low Dose Photoelectron Spectroscopy at BESSY II: Electronic Structure of Matter in its Native State. J. Electron Spectrosc. Relat. Phenom. 2018, 224, 68–78. 10.1016/j.elspec.2017.05.011.

[ref46] Zhang F.; Silver S. H.; Noel N. K.; Ullrich F.; Rand B. P.; Kahn A. Ultraviolet Photoemission Spectroscopy and Kelvin Probe Measurements on Metal Halide Perovskites: Advantages and Pitfalls. Adv. Energy Mater. 2020, 10, 1903252 10.1002/aenm.201903252.

[ref47] Malekan O.; Mohagheghi M. M. B.; Adelifard M. The Study of the Morphology and Structural, Optical, and J-V Characterizations of (CH_3_NH_3_PbI_3_) Perovskite Photovoltaic Cells in Ambient Atmosphere. Sci. Iran. 2021, 28, 1939–1952. 10.24200/sci.2021.55718.4372.

[ref48] De Angelis F. Modeling Materials and Processes in Hybrid/Organic Photovoltaics: From Dye-Sensitized to Perovskite Solar Cells. Acc. Chem. Res. 2014, 47, 3349–3360. 10.1021/ar500089n. 24856085

[ref49] Quarti C.; Mosconi E.; De Angelis F. Interplay of Orientational Order and Electronic Structure in Methylammonium Lead Iodide: Implications for Solar Cell Operation. Chem. Mater. 2014, 26, 6557–6569. 10.1021/cm5032046.

[ref50] Quarti C.; Mosconi E.; De Angelis F. Structural and electronic properties of organo-halide hybrid perovskites from ab initio molecular dynamics. Phys. Chem. Chem. Phys. 2015, 17, 9394–9409. 10.1039/C5CP00599J. 25766785

[ref51] Teng Q.; Shi T.-T.; Tian R.-Y.; Yang X.-B.; Zhao Y.-J. Role of Organic Cations on Hybrid Halide Perovskite CH_3_NH_3_PbI_3_ Surfaces. J. Solid State Chem. 2018, 258, 488–494. 10.1016/j.jssc.2017.10.029.

[ref52] Weinhardt L.; Ertan E.; Iannuzzi M.; Weigand M.; Fuchs O.; Bär M.; Blum M.; Denlinger J. D.; Yang W.; Umbach E.; et al. Probing Hydrogen Bonding Orbitals: Resonant Inelastic Soft X-ray Scattering of Aqueous NH_3_. Phys. Chem. Chem. Phys. 2015, 17, 27145–27153. 10.1039/C5CP04898B. 26417728

[ref53] Ekimova M.; Quevedo W.; Szyc L.; Iannuzzi M.; Wernet P.; Odelius M.; Nibbering E. T. J. Aqueous Solvation of Ammonia and Ammonium: Probing Hydrogen Bond Motifs with FT-IR and Soft X-ray Spectroscopy. J. Am. Chem. Soc. 2017, 139, 12773–12783. 10.1021/jacs.7b07207. 28810120

[ref54] Ekimova M.; Kubin M.; Ochmann M.; Ludwig J.; Huse N.; Wernet P.; Odelius M.; Nibbering E. T. J. Soft X-ray Spectroscopy of the Amine Group: Hydrogen Bond Motifs in Alkylamine/Alkylammonium Acid-Base Pairs. J. Phys. Chem. B 2018, 122, 7737–7746. 10.1021/acs.jpcb.8b05424. 30024171

[ref55] Reinholdt P.; Vidal M. L.; Kongsted J.; Iannuzzi M.; Coriani S.; Odelius M. Nitrogen *K*-edge X-ray absorption Spectra of Ammonium and Ammonia in Water Solution: Assessing the Performance of Polarizable Embedding Coupled Cluster Methods. J. Phys. Chem. Lett. 2021, 12, 8865–8871. 10.1021/acs.jpclett.1c02031. 34498464 PMC8450933

[ref56] Sun Z.; Chen M.; Zheng L.; Wang J.; Santra B.; Shen H.; Xu L.; Kang W.; Klein M. L.; Wu X. X-ray Absorption of Liquid Water by Advanced ab initio Methods. Phys. Rev. B 2017, 96, 104202 10.1103/PhysRevB.96.104202.

[ref57] Perdew J. P.; Ernzerhof M.; Burke K. Rationale for Mixing Exact Exchange with Density Functional Approximations. J. Chem. Phys. 1996, 105, 9982–9985. 10.1063/1.472933.

